# Thermosensory Transient Receptor Potential Ion Channels and Asthma

**DOI:** 10.3390/biomedicines9070816

**Published:** 2021-07-14

**Authors:** Oxana Yu. Kytikova, Tatyana P. Novgorodtseva, Yulia K. Denisenko, Denis E. Naumov, Tatyana A. Gvozdenko, Juliy M. Perelman

**Affiliations:** 1 Vladivostok Branch of the Far Eastern Scientific Center of Physiology and Pathology of Respiration, Institute of Medical Climatology and Rehabilitative Treatment, 690105 Vladivostok, Russia; nauka@niivl.ru (T.P.N.); karaman@inbox.ru (Y.K.D.); vfdnz@mail.ru (T.A.G.); 2 Far Eastern Scientific Center of Physiology and Pathology of Respiration, 675000 Blagoveshchensk, Russia; denn1985@bk.ru (D.E.N.); dncfpd@dncfpd.ru (J.M.P.)

**Keywords:** asthma, temperature, thermosensory transient receptor potential ion channels, genes

## Abstract

Asthma is a widespread chronic disease of the bronchopulmonary system with a heterogeneous course due to the complex etiopathogenesis. Natural-climatic and anthropogenic factors play an important role in the development and progression of this pathology. The reception of physical and chemical environmental stimuli and the regulation of body temperature are mediated by thermosensory channels, members of a subfamily of transient receptor potential (TRP) ion channels. It has been found that genes encoding vanilloid, ankyrin, and melastatin TRP channels are involved in the development of some asthma phenotypes and in the formation of exacerbations of this pathology. The review summarizes modern views on the role of high and low temperatures in airway inflammation in asthma. The participation of thermosensory TRP channels (vanilloid, ankyrin, and melastatin TRP channels) in the reaction to high and low temperatures and air humidity as well as in the formation of bronchial hyperreactivity and respiratory symptoms accompanying asthma is described. The genetic aspects of the functioning of thermosensory TRP channels are discussed. It is shown that new methods of treatment of asthma exacerbations caused by the influence of temperature and humidity should be based on the regulation of channel activity.

## 1. Introduction

Asthma is a widespread, chronic heterogeneous disease with a complex etiopathogenesis and constantly improving approaches to therapy. Nowadays, over 350 million people of all ages worldwide suffer from asthma, and about 350 thousand people per year die from the disease. The growing prevalence of this pathology among people of different age groups is of particular concern. Uncontrolled asthma that is diagnosed in 45% of patients with a high frequency of exacerbations and hospitalizations remains an important global health problem [1]. In Europe, about 15% of school-age children suffer from asthma; 5% of them have difficult-to-control asthma. According to the Global Burden of Disease Study, asthma occupies the 14th ranking place among the leading causes of disability. The global burden of asthma is associated with direct economic costs and indirect social and economic consequences [2]. Obviously, achieving asthma control and reducing exacerbation frequency in patients with the pathology are among the most pressing problems of our time.

According to current research, bronchospasm and asthma-like symptoms in asthma patients can occur under the influence of trigger factors, such as the combination of cold temperatures and high/low air humidity, as well as hot temperatures and high air humidity [3,4,5]. The contribution of high and low temperatures in airway inflammation in asthma as well as the main molecular mechanism underlying the disease have not yet been fully understood [5,6]. The high sensitivity of the bronchi to environmental factors is caused by impaired regulation of afferent nerves of the respiratory tract and chronic neurogenic inflammation [7]. Vagal sensory nerves, of which 75% are non-myelinated nociceptive C-fibers of the lungs, innervate the respiratory tract. In addition, nociceptor neurons are involved in the activation of the bronchoconstrictor mechanism in airway inflammation. The sensitivity of lung nociceptors is mediated by thermosensory channels, members of a subfamily of the transient receptor potential (TRP) ion channels [8]. The channels are considered as main sensors of physicochemical environmental stimuli involved in the regulation of body temperature [5]. The channels are activated by temperature changes [9]. TRP channels are expressed by neuronal cells and cells of the respiratory tract (bronchial epithelium and endothelium, smooth muscle cells, unmyelinated nociceptive C-fibers of the lungs) [10,11,12,13]. TRP channel activation in the nerve endings of the respiratory tract leads to the stimulation of protective reflexes, but under certain conditions, it can be the pathogenetic basis of asthma [12,13,14,15]. The role of TRP channels in asthma pathogenesis has been demonstrated [13,16,17,18,19,20,21], which makes them promising targets for disease therapy [12,13].

Asthma is initiated not only by the influence of natural and climatic factors but also by the interaction between these factors and the genetic component of the body. However, not all asthma patients react to changes in the temperature and humidity of inhaled air, which may be related to genetic factors [22]. Recently, there has been considerable interest in the genetic contribution to asthma pathogenesis [23,24,25,26,27,28,29,30,31,32]. Of particular interest is the identification of SNPs of TRP genes and their interaction with physicochemical environmental factors.

This review focuses on modern views on the role of high and low temperatures in airway inflammation in asthma. A brief description of the TRP superfamily and its subfamilies as well as a characterization of the TRP superfamily channels involved in the pathogenesis of bronchopulmonary diseases are given. The contribution of TRP channels (vanilloid, ankyrin-like, and melastatin) in response to exposure to high/low temperatures and air humidity as well as in the formation of bronchial hyperreactivity and respiratory symptoms accompanying asthma is discussed. The genetic aspects of the functioning of thermosensory TRP channels are described. It is shown that developing new methods of therapy for asthma exacerbations caused by the influence of temperatures and humidity should be based on regulating the activity of these channels.

Scientific publications on the topic that were published between December 2011 and December 2020 were obtained by searching PubMed© and Web of Science databases with the search terms “temperature, thermosensory receptors, transient receptor potential ion channels, genes, asthma”. Publications were limited to articles written in English. The review includes the sources of information that highlighted the structure and functioning of TRP ion channels in asthma and the genetic factors modulating their activity.

## 2. The Role of Natural-Climatic Factors in Asthma Pathogenesis

In recent years, a number of studies have shown that high and low air temperatures impact on the development and exacerbation of asthma [6,33,34]. In addition, the temperature effect is modulated by other environmental conditions (humidity, visibility, cloud cover, air pressure, wind speed, air pollution, etc.). For example, simultaneous exposure to low temperature and poor air quality is an important factor for the appearance of asthma symptoms [35]. Cold moist air induces more respiratory symptoms in asthma patients than cold dry air. Therefore, people suffering from this pathology should avoid adverse environmental conditions and should limit their outdoor activities during periods of extreme temperatures, as well as during periods of high humidity combined with low/high temperature and low humidity combined with low temperature [33].

Under normal conditions, nasal breathing partially compensates for the cold air exposure, and low airway symptoms at rest or during light exercise do not occur. Intensive exercise in cold weather generates only a short-term acute bronchospasm and cough symptoms in healthy people [36]. Nevertheless, the cold air is an important trigger factor for severe asthma in patients with cold airway hyperresponsiveness. Asthma patients exhibit an increase in the frequency of cold-air-induced symptoms by 50% compared with healthy subjects. The risk of developing respiratory symptoms provoked by cold air (shortness of breath, wheezing, sputum discharge) is more elevated in patients with asthma coexisting with allergic rhinitis [4]. M. D’Amato et al. have revealed that breathing of +20 °C air at 15 L/min reduces the temperature of the trachea proximal sections to 34 °C, while breathing the same air at 100 L/min decreases this temperature to 31 °C [37]. In addition to bronchoconstriction, cold air hyperventilation also brings about coughing. Cough and bronchospasm are independent reactions since pretreatment with salbutamol blocks bronchoconstriction but does not affect cold-air-induced cough. Additionally, cold air is dry, and consequently, cold air hyperventilation causes airway dehydration, leading to the release of mediators initiating bronchospasm [5].

The effect of conditioned cold air is also injurious to asthma patients. In particular, quick cooling of the indoor air without gradual adaptation to a temperature 2–3 °C lower than the outdoor temperature—and especially with humidity ranging between 40% and 60%—may give rise to asthma exacerbation over several hours or days [37].

There are few data on the negative impact of high ambient temperature on the respiratory tract [5,38,39,40]. Additionally, increased metabolic rate (physical activity) and difficult heat dissipation (for example, in a warm environment) are common causes of hyperthermia [40]. Junior M.A.V.C. et al. have found that exercise-induced bronchospasm is diagnosed in most asthmatic patients living in a hot dry climate, while only 10% of patients with rhinitis and 5% of healthy people living under the same conditions developed exercise-induced bronchospasm [38]. Non-myelinated nociceptive C-fibers of the lungs have been demonstrated to be activated when the intrathoracic temperature was elevated to 39.2 °C. Hot air hyperventilation induces the development of bronchoconstriction in asthma patients, and pretreatment with ipratropium bromide aerosol completely prevented bronchospasm in these patients [40]. Furthermore, hot air breathing provokes cough in these patients, which indicates damage to the respiratory nerves. Thus, hyperventilation with hot humid air raises the temperature of the airways and triggers bronchoconstriction in asthma patients by activating pulmonary C-fibers [40].

The association between asthma hospitalizations and air temperature is of interest. It has been established that in the cold season, temperature is inversely correlated with asthma hospital admissions [33]. Patients with poorly controlled asthma are more likely to exhibit cold-weather-related respiratory symptoms [35]. It is worth noting that the number of hospital admissions among adults depends not only on low temperature but also on high temperature, while this relationship is not observed among children under five years of age. In the hot season, a minimum number of asthma hospitalizations is observed at an ambient temperature of 27 °C; this parameter reaches the maximum at 30 °C and achieves a plateau in the temperature range 30–32 °C. High temperature can have a protective effect for adults but is dangerous for non-adults [41]. The amount of evidence suggesting a nonlinear association between air temperature and the number of asthma hospitalizations among children has been growing [6]. A stronger relationship between ambient temperature and repeat admissions of asthmatic children under five years of age compared with first admission cases has been shown. Repeat admissions demonstrated high sensitivity to both hot temperature in summer and low temperature in winter [34].

Thus, the combination of cold temperatures and high/low air humidity as well as the combination of hot temperatures and high air humidity lead to bronchospastic reactions and asthma-like symptoms (coughing, wheezing, shortness of breath) in asthma patients, thereby increasing the exacerbation frequency and reducing the control of the disease. It is known that thermosensory TRP channels are responsible for the reception of physicochemical environmental stimuli and the regulation of body temperature [5,8,9,42,43,44]. The impairment of their functioning plays an important role in asthma pathogenesis [16,17,18,19,20,21,45], which makes these channels promising targets for disease treatment [12,46].

## 3. Transient Receptor Potential Ion Channels

TRP channels belong to the voltage-gated ion channel superfamily (voltage-gated K^+^/Na^+^/Ca^2+^ channels, cyclic nucleotide-gated channels) localized mainly on the plasma membrane of cells [47]. The main difference between the TRP channel superfamily and other ion channel families is their activation in response to exogenous stimuli, such as temperature, chemicals, light, and sound [48,49]. The TRP superfamily consists of 28 channels grouped into 7 subfamilies, in each of which several subunits are distinguished [50,51]. The eighth subfamily was recently identified in yeast and named TRPY (Table 1).

**Figure 1:**
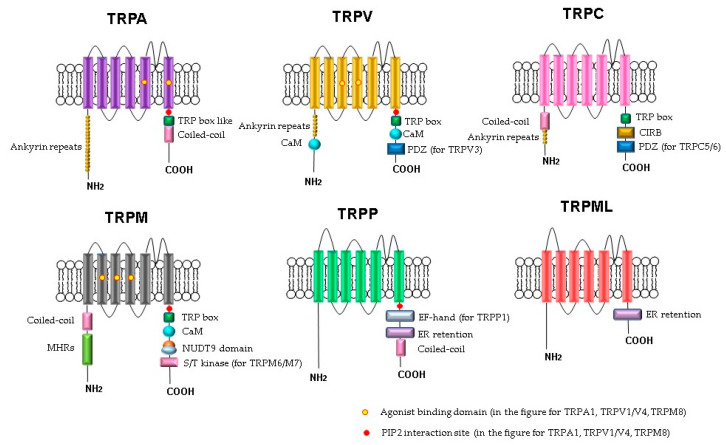
Schematic structure of TRP channels. Monomers of TRP channels consist of six transmembrane segments and a pore-forming return loop between S5 and S6. The intracellular N- and C-termini are distinguished in the length and domains. The TRP box is similar for TRPC, TRPV, TRPM, and a TRP box-like region for the TRPA1 channel. Coiled-coil domains (CC) are located in the C-terminus of TRPA and in the N-terminus of TRPC and TRPM. TRPM share melastatine high homology regions in their N-terminus, (MHRs). The TRPC subfamily of TRP channels has binding domains for calmodulin (CaM), inositol triphosphate receptor binding site (CIRB), and the PDZ-binding specific motif (PDZ). Substrates of NUDT9 are the adenosine 5′-diphosphoribose (ADPR) or the ADPR-2′-phosphate (ADPRP). NUDT9 domain is specific for TRPM2. TRPM6 and TRPM7 contain a serine/threonine kinase domain (S/T kinase). TRPP1 presents a calcium-binding motif (EF-hand), while TRPP and TRPML exhibit an endoplasmic reticulum retention (ER retention) domain. TRP channels are modulated by phosphatidylinositol-4,5-bisphosphate (PIP2).

The molecular understanding of TRP channels has been improved by structural biology methods [51]. Monomers of TRP channels consist of a six-pass transmembrane protein (S1–S6) and a re-entrant loop between S5 and S6, forming the pore or ion conduction pathway (Figure 1). The C- and N-termini of the TRP channels are in the cytoplasm. Some of the TRP channels contain ankyrin repeat domains in the N-terminus and a TRP-domain in the C-terminus. The number of ankyrin repeats in the ankyrin domains of the TRP subfamilies differs. For example, TRPC channels contain 3–4 repeats, TRPV channels have 6, TRPA has 14–18 repeats, and TRPN has 29. It is believed that the role of the ankyrin domain is in protein–protein interactions: channel tetramerization and binding to ligands and partner proteins. The ankyrin domains TRPV1, TRPV3, and TRPV4 bind ATP, which competes for binding with calmodulin, a protein that inhibits sensitization of TRPV receptors. The coiled-coil domain is another common motif in the proteins of the TRP family. It can be located both at the C-terminus and at the N-terminus and is involved in cytosolic interactions and binding of polyphosphates.

The operating principle of these receptors is the activation of cation influx in response to stimuli [47]. These channels are involved in the processes of apoptosis and proliferation, and they affect vascular tone. TRP channels are expressed primarily by neuronal cells. At the same time, the channels were also found on other cells, particularly in the respiratory tract (bronchial epithelium and endothelium, smooth muscle cells, non-myelinated nociceptive C-fibers of the lungs) [11,16,18,19,20,43,50,52,53,54,55,56,57,58,59] (Table 2).

## 4. The Disorder of TRP Channel Expression in Asthma Pathogenesis

The most studied TRP channels expressed in the airways are TRPA1, TRPV1, TRPV2, TRPV3, TRPV4, TRPC1, TRPC6, and TRPM8 [12,16,18,19,44,51,58,59,60,61,62,63,64,65,66,67,68,69,70,71,72,73,74,75,76,77] (Table 3).

Among TRP channels expressed in the airways, only TRPA1, TRPV1, TRPV2, TRPV3, TRPV4, TRPM3, and TRPM8 are thermosensory channels, which is why canonical receptors TRPC1 and TRPC1 are not described in this review.

The activation of TRPs in nerve endings of the airways leads to protective reflexes (cough, increasing mucus production and mucociliary clearance), but under certain conditions it would be liable to cause the development of asthma [13,15,17,42,47]. A study using a mouse asthma model has shown that high (40 °C) and low (10 °C) temperatures can exacerbate existing airway inflammation in the disease through the participation of TRP channels [5].

### 4.1. Vanilloid Receptors

The vanilloid TRP (TRPV) channels are predominantly localized in nociceptive neurons [52,56]. Additionally, a large amount of the receptors are present in sensory nerve fibers of the respiratory tract and on bronchial epithelial cells, mast cells, macrophages, and smooth muscle cells [54,55,65].

Bronchospasm and asthma-like symptoms developed in response to the action of cold air and high humidity are associated with the participation of TRPV1, TRPV4, and TRPV2 channels in osmoreception [53]. The alteration of intrabronchial osmolarity results in bronchial obstruction, mucociliary system dysfunction, and the activation of cough reflex [53,69]. Changes in the structure and functioning of the mucociliary system in turn contribute to airway hyperreactivity [15].

Particular attention is paid to TRPV1-mediated induction of cough as the result of inhalation of irritating environmental reagents and in asthma [15,42,53,66]. The release of pro-inflammatory mediators that activate or sensitize TRPV1 channels (prostaglandin, bradykinin, lipoxygenase metabolites) is accompanied by the development of cough [15]. It is known that a decreased pH in diseased airways also causes the activation of TRPV1 channels [13,47]. Considering the fact that the prophylactic administration of antagonists of TRPV1 (AMG9810) and LPA (BrP-LPA) receptors prevents the development of bronchoconstriction, vanilloid receptors can be an important target for asthma therapy. TRPV1 channels are widely expressed in the nasal mucosa. TRPV1 channel overexpression has been reported in patients with idiopathic rhinitis; additionally, it has been shown that capsaicin blocks the nociceptive TRPV1 signaling pathway in the mucosa. An increased expression of TRPV1 and TRPV2 proteins in lung tissue has been found.

It has been revealed that TRPV3 channels are overexpressed in non-small-cell lung cancer and their expression correlates with tumor progression. TRPV3 channel activation can promote lung cancer cell proliferation [55].

TRPV4 channels are expressed in smooth muscles, fibroblasts, macrophages, submucous glands, endothelial cells, epithelial cells of the trachea, bronchi, and alveoli [16,56,68,69]. The TRPV4 channel is currently considered as a regulator of embryonic lung development. Under ex vivo conditions, the TRPV4 channel has been shown to be involved in airway morphogenesis [16]. This channel regulates smooth muscle contractility, thereby affecting pulmonary vascularization [69]. It has been established that reactive oxygen species (hydrogen peroxide (H_2_O_2_)) activate TRPV4 channels through the Fyn kinase-mediated mechanism. The interaction of TRPV4 channels and reactive oxygen species impairs the barrier function of the lungs [56].

### 4.2. Ankyrin-Like Receptors

The TRP ankyrin 1 (TRPA1) channels were found in T cells, B cells, mast cells, the dorsal root ganglia, trigeminal ganglia, and nociceptive C-fibers of the lungs. The TRPA1 channel was first cloned from cultured fibroblasts and further found in fibroblasts cell lines and human lung epithelial cells [67]. The channel is activated by cold, menthol, icilin, hypoxia, and hyperoxia [11]. Along with TRPV1, TRPA1 plays a role in triggering chronic inflammation of the respiratory tract, primarily as a protective mechanism [42,61,62,63], and it is also involved in the activation of the cough reflex [34]. Thus, cold air induces an airway inflammatory reaction and bronchial remodeling by changing TRPA1 expression [78]. In the study by C. Du et al. mice were exposed to different temperatures (26 °C and 10 °C cycles) during the 21-day period. As a result, a TRPA1-mediated temperature-dependent aggravation of airway inflammation was observed [61]. The role of TRPA1 in the activation of sensory nerves of the lungs and the development of bronchoconstriction has been established for both allergic [61] and non-allergic [79] asthma. TRPA1 appears to be important in childhood asthma [27].

TRPA1 and TRPV1 channels are often co-expressed and functionally interact [62,63]. TRPV1 also can form complexes with other subunits of the TRP family, including TRPV3 [53]. TRPA1 and TRPV1 are co-expressed in non-myelinated nociceptive C-fibers of the lungs. Since these channels are sensitive to endogenous inflammatory mediators, they are simultaneously activated in airway inflammation [34]. The studies by Lu-Yuan Lee et al. have shown a potentiating effect induced by simultaneous activations of TRPA1 and TRPV1 by their respective selective agonists, allyl isothiocyanate, and capsaicin [63]. This effect was absent when the agonists were replaced by other chemical activators of neurons. In addition, synergism depended on extracellular Ca^2+^ levels [63]. These data suggest the importance of TRPA1–TRPV1 interaction for regulating functioning and excitability of sensory neurons of the lung in airway inflammation.

### 4.3. Melastatin Receptors

The TRP melastatin (TRPM) family is subdivided into four groups: TRPM1/3, TRPM2/8, TRPM4/5, and TRPM6/7 [50,57,73]. One of the actively studied sensory receptors of the TRP family is the TRPM8 receptor, which is activated by cold temperatures and cooling agents, such as menthol, icilin, and eucalyptol [19,44,58,59]. The activation of TRPM8 by cooling compounds blocks the transmission of pain signals [76]. In addition to the above-mentioned role, TRPM8 is involved in the processes of thermoregulation in mammals. According to recent studies, TRPM8 is localized in airway epithelial and smooth muscle cells as well as in trigeminal and vagus nerves innervating the respiratory tract. It has been revealed that TRPM8 expression is increased in the respiratory tract in patients with asthma [20]. Cold-air-caused activation of TRPM8 leads to the elevation of expression of cytokines, chemokines, and mucus hypersecretion, followed by bronchial remodeling [58,59,78]. Moreover, the role of TRPM8 in the induction of inflammation, mucus hypersecretion, and airway remodeling in asthma patients even in the absence of the influence of irritants has been reported [20,22]. Experimental studies on asthma have confirmed that cold air can cause inflammatory responses via theTRPM8-mediated NF-κB signaling pathway in epithelial cells of the respiratory tract [18,19]. At the same time, the TRPM8 antagonist PF-05105679 does not change body temperature but causes a sensation of heat in the oral cavity [76]. It has been established that sputum TRPM8 protein level is higher in asthma patients than in healthy subjects and significantly higher in bronchodilator-sensitive asthmatics than in nonresponders [72]. TRPM8 channel is abundantly expressed in the nasal subepithelium and in idiopathic rhinitis. It has been shown that a decrease in TRPM8 channel expression after capsaicin treatment improves the nasal mucosa hyperreactivity. Obviously, TRPM8 could be a molecular target for asthma therapy [77].

Therefore, the development of bronchospasm and asthma-like symptoms in response to the action of hyper- and hypoosmolar exogenous stimuli is mediated by the vanilloid receptors, such as TRPV1, TRPV4, and TRPV2. Cold and hot air induce inflammation and bronchial remodeling through the expression of ankyrin-like (TRPA1) and melastatin (TRPM8) receptors. Obviously, new methods for the treatment of asthma exacerbations caused by the influence of temperatures and humidity should be based on regulating the activity of TRPA1, TRPM8, and TRPV channels [64]. Currently, a number of compounds modulating the activity of TRP channels (TRPV1, TRPV3, TRPV4, TRPA1, TRPM8) have successfully passed clinical trials [46,77]. However, several TRPV1 antagonists were excluded from clinical trials because of the hyperthermic reaction they caused.

It should be noted that the heterogeneity of respiratory reactions in response to physicochemical environmental factors in asthma patients may be due to a genetic component [75]. In particular, polymorphisms of genes of vanilloid, ankyrin-like, or melastatin receptors can affect their functions and promote the development of various phenotypes of asthma (severe asthma, childhood asthma, etc.), which require further study.

## 5. Genetic Variability of TRP Channels in Asthma Pathogenesis

A number of studies have reported that the genetic contribution to the risk for asthma is estimated as 55–74% in adults and 90% in children [80,81]. The concordance for this pathology—cases of asthma in children born to mothers with the disease and cases of asthma in several generations of the same family—has been described [82,83]. The existence of various forms of a genetic trait in the body indicates gene polymorphisms. Single-nucleotide polymorphisms (SNP) are more common. Variations on chromosome 17q21 were associated with asthma [84]. The most significant association was revealed between asthma and rs2549003 SNP of *IRF1*, which was related to this disease in men [85]. GWAS results have shown that genes associated with mucosal immunity and epithelial physiology are involved in the pathogenesis of early asthma [86]. Unfortunately, information on the influence of demographic factors, socioeconomic status, educational level, and many other risk factors on the development of asthma is limited [24]. In particular, there is not enough research on the interactions between genes, environmental exposure, and risk for asthma developing. In this regard, research of the relations between genes and the environment could improve understanding of basic mechanisms of the pathogenesis of the pathology [28]. Available data on the genetic variability of TRP channels in asthma pathogenesis are presented below.

### 5.1. Genes of Vanilloid Receptors in Asthma Pathogenesis

A new-generation sequencing panel (NGS) made it possible to identify 140 chromosome loci where the nucleotides deviated from the reference sequence, GRCh37 hg19, including three genes (*TRPV1*, *LTB4R*, and *LTB4R2*) [87]. An increased expression of the *TRPV1* and *TRPV2* genes in lung tissue has been found [67]. Taking into account the central role of TRP channels in the activation of the cough reflex, changes in the genes encoding these channels may be associated with a cough caused by irritating substances. It has been shown that missense SNP *TRPV1* rs224534 (p.Thr469Ile) increases the susceptibility to cough in smokers and in individuals exposed to air irritants [87].

**Figure 2:**
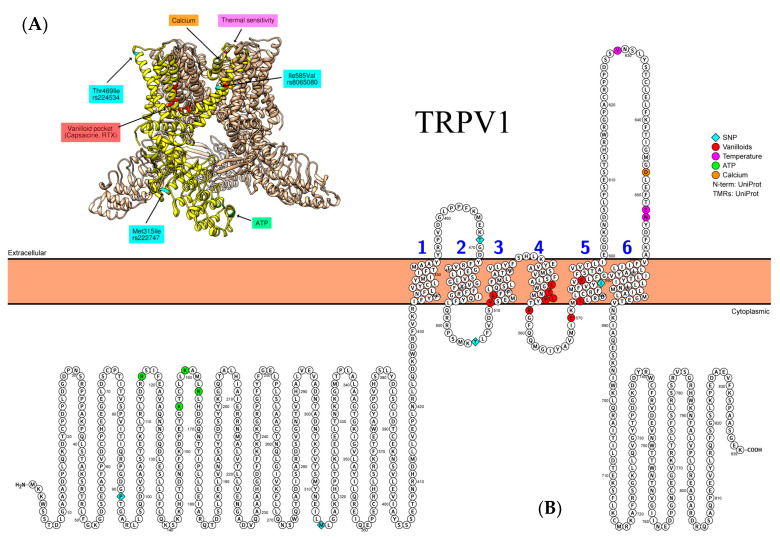
Representative structure of TRPV1 (PDB ID: 3J5P) channel. (**A**) Individual subunit is highlighted in yellow. Not all known binding sites/SNPs are shown due to limited coverage of the currently available PDB models. Molecular graphics and analyses performed with UCSF Chimera 1.15, developed by the Resource for Biocomputing, Visualization, and Informatics at the University of California, San Francisco, with support from NIH P41-GM103311 (here and in the Figure 3, Figure 4 and Figure 5). (**B**) Relevant amino acid substitutions for SNPs in the coding regions of TRPV1: rs222749 (p.Pro91Ser) and rs222747 (p.Met315Ile) in the N-terminus; rs224534 (p.Thr469Ile) and rs17633288 (p.Thr505Ala) in the S2; and rs8065080 (p.Ile585Val) in the S5. The figure was created in the Protter platform https://wlab.ethz.ch/protter/start/ (accessed on 20 May 2021) (here and in the Figure 3, Figure 4 and Figure 5).

It has been found that a mutation of the *TRPV1* gene is closely related to the development of asthma in children [88,89,90]. Chen C.L. et al. have revealed that *TRPV1* gene expression level and mutation rs4790522 located in 3′-UTR are the main risk factors for childhood asthma [89]. Recent research results have shown that the *TRPV1* p.Ile585Val SNP (rs8065080) is associated with a lower risk for developing cough and shortness of breath in asthmatic children by blocking of the channel signal transduction [90]. This loss-of-function *TRPV1* variant also correlated with a decrease in cough and wheezing severity in asthma patients. At the same time, the presence of rs8065080 and synonymous SNP rs222748 (p.His147=) of *TRPV1* gene is unrelated to non-specific chronic cough in children [88] (Figure 2). In the study by Liveiro F. et al., only combined but not individual SNPs in the coding region of *TRPV1* were associated with altered cough sensitivity to capsaicin [91]. The effect was established for the following combinations: I315M + P91S, I585V + P91S, I315M + I585V + T469I, I315M + I585V + P91S and I315M + I585V + T469I + P91S. The effect of T505A was not assessed, as it was not revealed in the studied population. With regard to I585V, it is remarkable that this SNP indeed is located in close proximity to Phe587 residue, which is important for vanilloids binding, so its functional impact is structurally justified (Figure 2). Missense SNPs do not necessarily affect only protein structure and binding kinetics—instead, they may influence protein expression. It has been established that P91S and I315M SNPs increase the expression of TRPV1 protein, whereas the expression of mRNA remains relatively unchanged [92]. According to Deering-Rice et al., I315M and T469I also increase the sensitivity of TRPV1 receptors to capsaicin and coal fly ash (CFA) [93]. On the contrary, the I585V variant is less responsive to CFA due to reduced translation of the protein.

**Figure 3:**
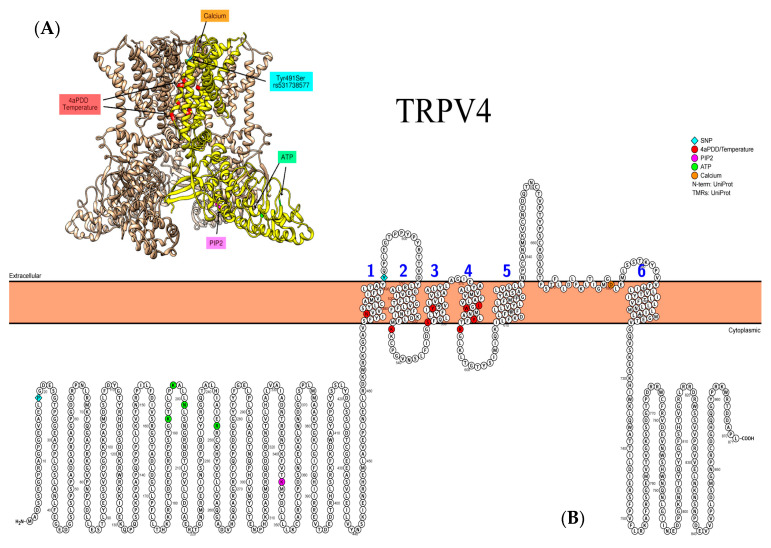
Representative structure of TRPV4 (PDB ID: 6BBJ) channel. (**A**) Individual subunit is highlighted in yellow. Not all known binding sites/SNPs are shown due to limited coverage of the currently available PDB models. (**B**) Relevant amino acid substitutions for SNPs in the coding regions of TRPV4: rs3742030 (p.Pro19Ser) and rs531738577 (p.Tyr491Ser).

The *TRPV4* gene SNP rs6606743 located in the 5′-upstream region has been revealed to make a significant contribution to the development of osmotic airway hyperresponsiveness in patients with uncontrolled asthma [75] (Figure 3). Another SNP of *TRPV4*—a missense variation rs3742030 (p.Pro19Ser)—demonstrated a lack of association with childhood asthma and cough or current wheezing as markers of active disease [90]. Nonetheless, Zhu G. et al. reported a significant association of this SNP with COPD or pulmonary function traits [94]. There are conflicting results regarding functional consequences of Pro19Ser amino acid substitution [95]. It was considered as loss-of-function under activation by mild hypotonicity or epoxyeicosatrienoic acid. However, a gain-of-function effect was observed when the channel was activated by diesel exhaust particles or its organic extract [96].

TRPV2 expression in sensory neurons and its heat-mediated activation suggest the involvement of this channel in nociception. However, deletion of the *TRPV2* gene does not alter thermal perception in mice [67]; therefore, the role of TRPV2 in thermogenesis is still controversial. At the same time, patients with asthma exhibited an increased mRNA expression of *TRPV2* in lung tissues or cells [88]. In general, the role of vanilloid receptor genes in asthma pathogenesis is incompletely studied.

### 5.2. Genes of Ankyrin-Like Receptors in Asthma Pathogenesis

It should be noted that not only features of TRPA1 channel expression in asthma but also the impact of variations in the *TRPA1* gene on the development and course of asthma are still far from clear.

**Figure 4:**
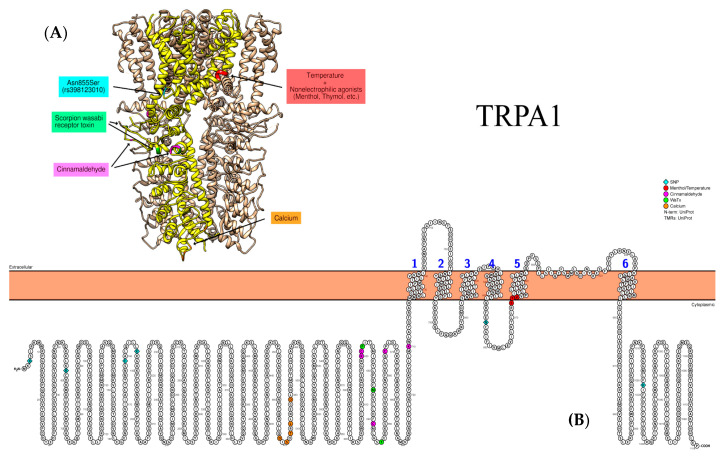
Representative structure of TRPA1 (PDB ID: 3J9P) channel. (**A**) Individual subunit is highlighted in yellow. Not all known binding sites/SNPs are shown due to limited coverage of the currently available PDB models. (**B**) Relevant amino acid substitutions for SNPs in the coding regions of TRPA1: rs13268757 (p.Arg3Cys), rs16937976 (p.Arg58Thr), rs920829 (p.Glu179Lys), and rs7819749 (p.Lys186Asn) in the N-terminus; rs398123010 (p.Asn855Ser) in the S4–S5 linker; and rs959976 (p.His1018Arg) in the C-terminus.

An increased *TRPA1* gene expression in the lung tissue and interleukine-4 (IL-4) level in the supernatant of the lung homogenate and levels of IL-13, substance P, prostaglandin D2, the nerve growth factor in bronchoalveolar lavage fluid, and the supernatant of lung homogenate have been reported in OVA-sensitized mice under exposition with common pollutant—trimellitic anhydride [26]. Evidence for the relationship between *TRPA1* SNPs in coding (rs920829, rs959976) and non-coding regions (rs959974, rs1384001, rs7010969, rs3735945, rs920829, and rs4738202) and asthma developing in children aged 7–8 years has been presented [27] (Figure 4). By contrast, these SNPs do not contribute to a genetic predisposition to asthma among adults. Only the presence of 3′-UTR rs6996723 SNP has been associated with a high risk of the disease among people over 18 years of age [97] (Figure 4).

Reese R.M. et al. has defined that *TRPA1* gene blocking under experimental conditions causes a lower the number of immune cells recruited into the respiratory tract in rats in response to an asthmatic stimulus [64]. Additionally, it has been shown that TRPA1 is an essential factor in the development of asthma/allergic hypersensitivity in mice, while Arg3Cys and Arg58Thr polymorphisms correlate with worse asthma control in children. These variants along with His1018Arg demonstrated enhanced activation by CFA particles, while Glu179Lys, Lys186Asn, and Asn855Ser decreased the response to CFA [98]. In contrast to these results, the study by Kremeyer B. et al. demonstrated gain-of-function properties of Asn855Ser SNP [99]. The mutant variant enhanced TRPA1 response to cooling and response to cinnamaldehyde and other agonists, such as 4-hydroxynonenal, allyl isothiocyanate or menthol. In addition, the same study showed that Asn855Ser mutation causes autosomal-dominant familial episodic pain syndrome. Furthermore, in accordance with previous findings, Gupta R. et al. established a diminished inhibition of Asn855Ser variant of TRPA1 by HC-030031 antagonist [100].

### 5.3. Genes of Melastatin Receptors in Asthma Pathogenesis

**Figure 5:**
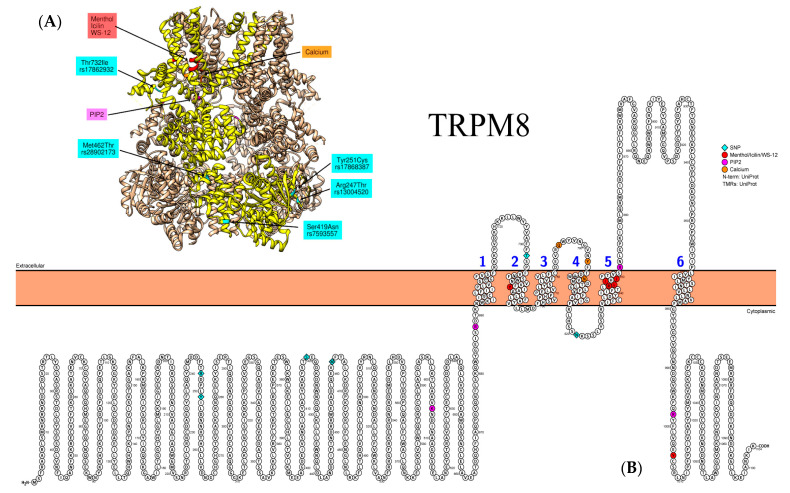
Representative structure of TRPM8 (PDB ID: 6BPQ) channel. (**A**) Individual subunit is highlighted in yellow. Not all known binding sites/SNPs are shown due to limited coverage of the currently available PDB models. (**B**) Relevant amino acid substitutions for SNPs in the coding regions of TRPM8: rs13004520 (p.Arg247Thr), rs17868387 (p.Tyr251Cys), rs7593557 (p.Ser419Asn), and rs28902173 (p.Met462Thr) in the N-terminus; rs17862932 (p.Thr732Ile) in the S2 linker; and rs28902201 (p.Asn821Ser) in the S4–S5 linker.

TRPM8 plays a crucial role in the response of the lungs to a cold stimulus [19,44]. In humans, the *TRPM8* gene is localized to chromosome 2q37. Recent studies have reported the existence of TRPM8 channel isoforms encoded by 33 alternative mRNAs [73]. TRPM8 is involved in the innate immune response of airway epithelial cells. It has been found that *TRPM8* mRNA expression by human bronchial epithelial cells strongly correlated with the synthesis of IL-25 and the thymic stromal lymphopoietin of these cells [72]. The role of synonymous SNP rs11562975 (p.Leu250=) of the *TRPM8* gene in the formation of cold-induced airway hyperresponsiveness in asthma patients has been established [75]. The contribution of *TRPM8* rs11562975 SNP to the reduction in pulmonary function and the development of severe asthma have been demonstrated. The knockdown of TRPM8 attenuated cold-related inflammation neutralized the imbalance between Th1 and Th2, as well as had a positive effect on airway remodeling [19]. The effects of *TRPM8* polymorphism also depend on climatic conditions [101]. Currently, there are no convincing data regarding functional consequences of *TRPM8* missense SNPs (rs13004520 (p.Arg247Thr), rs17868387 (p.Tyr251Cys), rs7593557 (p.Ser419Asn), rs28902173 (p.Met462Thr), rs17862932 (p.Thr732Ile), rs28902201 (p.Asn821Ser)) or their association with certain disorders, including respiratory diseases [22,101,102] (Figure 5).

The *TRPM3* gene encodes different TRPM3 isoforms [103]. The *TRPM3* rs10780946 intronic variant is associated with the susceptibility of aspirin-exacerbated respiratory disease (AERD). The respiratory disease is the combination of asthmatic rhinosinusitis and polyposis; taking aspirin or other non-steroidal anti-inflammatory drugs worsens asthma symptoms. AERD pathogenesis remains unclear, but it is known that genetic and environmental factors contribute to the development of this pathology. Therefore, the genes of vanilloid and ankyrin-like and melastatin receptors are involved in the development of uncontrolled and childhood asthma, and this issue remain to be elucidated.

### 5.4. Prediction of Functional Impact of Nonsynonymous TRP Genetic Variants

Considering that not all known missense variations in the described TRP genes were properly characterized in terms of their influence on protein function as well as in order to facilitate the study of these SNPs, we analyzed possible functional consequences of amino acid substitutions using three of the most known web tools: PROVEAN [104], SIFT [105], and PolyPhen-2 [106]. A summary of the predicted effects for missense SNPs of *TRPV1*, *TRPV4*, *TRPA1*, and *TRPM8* is presented in Table 4.

Despite the useful results for some variations that are also in agreement with experimental data (rs224534 (p.Thr469Ile), rs13268757 (p.Arg3Cys), rs920829 (p.Glu179Lys)), generally, all the mentioned software seems to suffer from relatively low sensitivity, as the known deleterious effects for many other SNPs remained undetected. Nevertheless, three SNPs were consistently found as damaging (*TRPV4* rs531738577 (p.Tyr491Ser), *TRPM8* rs28902173 (p.Met462Thr), and rs17862932 (p.Thr732Ile)), though their effects were never previously studied in vitro or in vivo.

## 6. Endogenous and Exogenous Activators of Thermosensory TRP Channels

Thermosensor TRP channels (TRPV1, TRPV2, TRPV3, TRPV4, TRPM3, TRPM8, TRPA1) are members of the subfamily of TRP channels that are activated when the ambient temperature changes [9,11,16,19,43,44,51,52,53,54,55,56,57,58,59]. It should be noted that these channels are also activated under the influence of other exogenous and endogenous mediators. Nowadays, there are more than 60 structures with an atomic resolution of the TRPA1, TRPV1, TRPV2, TRPV3, TRPV4, TRPC5, TRPC6, and TRPM8 channels that have been deposited in the Protein Data Bank (www.rcsb.org (accessed on 20 May 2021)). PDB codes of TRPA1, TRPV1/V2/V3/V4, TRPVC5/C6, and TRPM8 channels and their ligands are presented in Table 5.

The activation of TRPA1 occurs at temperatures below 8 °C; TRPM8 is initiated within a temperature range between 8 °C and 28 °C; TRPV3 and TRPV4 are responsible for temperature perception in a temperature range of 27 °C to 35 °C; TRPV1 and TRPM3 are triggered at temperatures more than 42 °C; the temperature range for TRPV2 activation is above 52 °C [19,43,44,53,55,56,57]. Thus, TRPVs are heat-activated channels [16]; TRPA1 and TRPM8 are cold-activated channels [11,19,37,39,43,58,59]. Exposure to high and low temperatures is accompanied by TRP-mediated pain. Some thermosensory channels are activated by molecules from spices, such as garlic (allicin), chili pepper (capsaicin), wasabi (allyl isothiocyanate), as well as menthol, cannabinoids, cinnamaldehyde, camphor, stevia, peppermint, and cooling agents. TRP channel activators are also reactive oxygen species, reactive nitrogen species, bradykinin, prostaglandin E2, formaldehyde, and tear gases. There are channels acting as sensors for osmotic pressure, volume, tension, and vibration.

Therefore, TRP channels are involved in many processes and are activated by a variety of stimuli.

## 7. Thermosensory TRPs as Potential Drug Targets in Asthma

Currently, it has been established that a number of thermosensory TRPs are potential pharmacological targets for asthma [107]. Antagonists of TRP channels tested in experimental and clinical trials in asthma are summarized in Table 6.

### 7.1. TRPA1

TRPA1 have become one of the most promising targets for drug development in chronic cough, asthma, chronic obstructive pulmonary disease (COPD), and allergic rhinitis [108]. According to preliminary data, TRPA1 antagonists have no effect on thermal sensitivity; therefore, they do not cause dysregulation of body temperature, unlike TRPV1 (hyperthermia) or TRPM8 (hypothermia) antagonists. However, the efficacy of TRPA1 antagonists in animal models of bronchopulmonary pathology is not evidence of their unequivocal benefit for the treatment of human respiratory diseases. The administration of TRPA1 antagonists has been shown to reduce late symptoms of asthma in experimental animals [79]. It has been found that citric-acid-induced cough is suppressed by the selective TRPA1 antagonist, GRC 17536, in guinea pigs [108]. However, capsazepine, which is a TRPV1 antagonist and a desensitizing TRPA1 agonist, has no significant effect on cough [109]. Aparici M. et al. have reported antitussive action of the TRPA1 antagonist compound A in guinea pigs [110].

According to recent data, persistent cough in guinea pigs is attenuated by the selective TRPA1 antagonist HC-03003. There are a number of experimental research studies devoted to the study of the effect of the selective TRPA1 blocker HC-030031 on bronchopulmonary inflammation [111]. It has been demonstrated that HC-030031 reduces TRPA1-mediated calcium influx in TNF-α-treated lung fibroblasts, decreases the level of intracellular ROS, and blocks the MAPK/NF-κB signaling pathway [112].

TRPA1 has been found to contribute to E-cadherin and β-catenin dysfunction in TDI-induced asthma. The decrease in E-cadherin and β-catenin levels observed after exposure to TDI was inhibited by both the TRPV4 blocker (GSK2193874; 5 and 10 mg/kg) and the TRPA1 blocker (HC030031; 10 and 20 mg/kg) [113]. In addition, these inhibitors also suppressed serine phosphorylation of glycogen synthase kinase 3β and tyrosine phosphorylation of β-catenin and nuclear transport of β-catenin.

It has been shown that the selective TRPA1 antagonist AP-18 attenuates cough stimulated by the TRPA1 agonist (AITC; 10 mM) in guinea pigs. Cough caused by cinnamaldehyde (10 mM) was partially inhibited by AP-18 as well as by the combination of AP-18 and I-RTX (the TRPV1 antagonist) [114]. However, it has been noted that AP-18 has limited value for studying TRPA1-mediated responses in smooth muscles and should be used with caution due to possible non-specific effects [115].

The TRPA1 antagonists CB-189625 and GRC17356 are undergoing phase I clinical trials for the fight against chronic cough in asthma patients [116].

### 7.2. TRPV1

Despite a large number of studies on TRPV1 functions, there are no drugs targeting this channel [117,118]. The TRPV1 antagonists induce hyperthermia, which limits their use in clinical practice [116]. The clinical use of the TRPV1 antagonist JNJ17203212, which suppressed capsaicin-induced cough and was highly selective and well-tolerated, has been halted. A promising antagonist is JNJ39729209, which suppresses capsaicin-induced cough in guinea pigs but causes a slight increase in temperature (only 1 °C). Effective inhibitors of TRPV1 include SB-705948 and XEN-D0501 [119]. SB-705498 is a non-hyperthermia inhibitor, but it is not very effective in suppressing capsaicin-induced cough. Moreover, it does not affect idiopathic cough. Comparative efficacy of XEN-D0501 and SB-705498 in refractory chronic was carried out in preclinical studies [120]. Overall, the XEN-D0501 was more efficient than the SB-705498. Preclinical studies have shown that this inhibitor does not induce hyperthermia. XEN-DO501 is currently undergoing phase II clinical trials (double-blind, randomized, placebo-controlled) in patients with COPD.

The use of inhibitors SB-705498 and PF-04065463 has been reported to suppress ovalbumin-induced airway hypersensitivity to histamine in guinea pigs [121]. At the same time, exposure to SB-366791 did not influence eosinophil infiltration in allergic asthma in mice [122]. In general, TRPV1 antagonists have not demonstrated a pronounced positive effect in chronic cough and the diseases of the bronchopulmonary system associated with it [116,120,123].

### 7.3. TRPM8

Modern studies have highlighted an important role of TRPM8 in the immune response of airway epithelial cells [72]. According to existing data, the use of the TRPM8 antagonist BCTC (10 μM) reduces menthol-induced the TRPM8 mRNA and protein expression on BEAS2B human bronchial epithelial cells, the thymic stromal lymphopoietin (TSLP) level, and IL-25 mRNA. As has been experimentally proved, the TRPM8 channel inhibitor AMTB (N-(3-aminopropyl)-2-[(3-methylphenyl)methoxy]-N-(2-thienylmethyl)benzamide hydrochloride) suppresses the T cell activation caused by various stimulants, along with TRPV1 and TRPV4 inhibitors [124]. Another TRPM8 antagonist is JNJ41876666. Maher S. et al. have found that the TRPM8 agonist WS3 and menthol activate the vagus nerves in humans and mice [125]. Pre-incubation with menthol inhibited capsaicin-induced activation of the vagus nerve. This effect was not suppressed by JNJ41876666 [125]. The endocannabinoids N-arachidonoylethanolamide (AEA or anandamide) and 2-arachidonoylglycerol (2-AG) can interact directly with cannabinoid receptors CB1 and CB2 localized in the bronchi of mice, which may be interesting in terms of studying their therapeutic role in asthma [126,127]. Cannabinoid activity is mediated by TRPV1-TRPV4, TRPA1, and TRPM8 [128]. Nevertheless, cannabinoids act on the first five mentioned channels as agonists, and only for TRPM8 are they antagonists. N-arachidonyl dopamine (NADA) and anandamide (AEA) were the first endogenous TRPM8 antagonists discovered. The activity and therapeutic potential of N-acylamides in asthma remain to be investigated.

## 8. Conclusions

Current epidemiological studies convincingly indicate that temperature is a trigger for the development of asthma and its exacerbations, thereby reducing disease control. However, the role of high and low temperatures in the pathogenesis of chronic airway inflammation and the molecular mechanism underlying this process are far from being completely understood. The combined exposure to high or low temperatures and humidity generates bronchospasm and asthma-like symptoms in asthma patients through transient receptor potential ion channels participating in the reception of physical and chemical environmental stimuli. The development of bronchospastic reaction and asthma-like symptoms in response to a hyper- or hypoosmolar exogenous stimulus is mediated by vanilloid receptors. Cold and hot air provokes inflammation and bronchial remodeling by the expression of ankyrin-like and melastatin receptors. Obviously, the development of new methods for treating asthma exacerbations caused by the influence of temperatures and humidity should be based on regulating the activity of these channels. The genes of vanilloid, ankyrin-like, and melastatin TRP channels are involved in the formation of some asthma phenotypes and the development of exacerbations of this pathology; however, this issue requires further study. Changes in genes encoding vanilloid receptors are believed to associate with irritant-induced cough, but the role of the *TRPV* gene in thermogenesis remains controversial; the role of these receptor genes in asthma pathogenesis also should be investigated further. The association of genetic variations in the *TRPA* gene with the development and course of asthma is also insufficiently studied. The role of *TRPM* gene polymorphism in the formation of cold airway hyperreactivity has been established. *TRPM* gene knockdown attenuates cold-induced inflammation, reduces Th1/Th2 imbalances, and has a positive effect on airway remodeling. The phenotypic realization of *TRPM* polymorphism depends on climatic conditions. Further research on the interactions between *TRP* gene polymorphism and physicochemical environmental factors will allow us to elucidate the mechanisms underlying asthma pathogenesis and to detect new targets for asthma treatment.

## Figures and Tables

**Table 1:** TRP channel superfamily.

TRP Channel Subfamily	Subfamily Subunits
canonical receptors	TRPC 1–7
vanilloid receptors	TRPV1–6
polycysteine receptors	TRPP1–3
mucolipin receptors	TRPML1–3
ankyrin-like receptors	TRPA1
melastatin receptors	TRPM1–8
Drosophila NOMPC in mammals	TRPN

**Table 2:** Tissue or cells expressing thermosensory TRP channels.

TRP Channels	Tissue or Cells	References
TRPA1	T cells B cells peptidergic and non-peptidergic neurons myelinated Aβ-fibers epithelial cells melanocytes mast cells fibroblasts enterochromaffin cells lung cells	[11,43,48,49]
TRPV1	epithelial cells melanocytes mast cells fibroblasts enterochromaffin cells lung cells airway-specific neurons C fibres Aδ-fibres central nervous system (neurons, glial cells, astrocytes) pulmonary arteries aorta gastrointestinal tract	[52,53,54]
TRPV2	neuronal and non-neuronal tissues pulmonary arteries aorta gastrointestinal tract	[52]
TRPV3	epithelial cells of skin oral cavity gastrointestinal tract tongue dorsal root ganglion trigeminal ganglion spinal cordbrain lung cells neuronspulmonary arteries aorta	[52,55]
TRPV4	gastrointestinal tract prostate smooth muscle cells vascular endothelium pulmonary arteries aorta epidermal keratinocyte cells fallopian tubes epithelial cells of the human cornea insulin secreting β cells of the pancreas urothelial cells in the renal pelvis ureters urethra urinary bladder enterocytes and enteroendocrine cells fibroblasts macrophages submucosal glands lung cells central nervous system (neurons, glial cells, astrocytes) peripheral nervous systems	[16,47,52,56]
TRPM3	lung cells pulmonary arteries aorta cancerous tissues dorsal root ganglia cardiomyocytes insulin secreting β cells of the pancreas	[50,57]
TRPM8	lung cells pulmonary arteries aorta afferent neurons in cold-sensitive afferents expressed in the upper and lower airways nasal trigeminal neurons retrogradely labelled jugular neurons cancerous tissues	[19,20,44,50,58]

**Table 3:** Characterization of the superfamily of TRP channels involved in the pathogenesis of bronchopulmonary diseases.

TRP Channels	Lung Cells	Functions	Diseases	References
TRPA1	bronchial epithelial cells endothelium airway smooth muscle cells fibroblasts alveolar type 1/type 2	inflammation, airway hyperreactivity and remodeling, bronchoconstriction, cough reflex	asthma, chronic obstructive pulmonary disease, cystic fibrosis, cough, laryngeal obstruction, rhinitis	[60,61,62,63,64]
TRPV1	bronchial epithelial cells endothelium airway smooth muscle cells airway-specific neurons C fibres Aδ-fibres	inflammation, airway hyperreactivity and remodeling, bronchoconstriction, cough reflex	chronic obstructive pulmonary disease, asthma, idiopathic pulmonary fibrosis, chronic idiopathic cough, rhinitis, dyspnea	[53,60,62,65,66]
TRPV2	alveolar macrophages	inflammation, airway hyperreactivity and remodeling, bronchoconstriction, cough reflex	asthma, cancer, chronic obstructive pulmonary disease	[26,67]
TRPV3	bronchial epithelial cells	inflammation, airway hyperreactivity and remodeling	pneumotoxicity	[55]
TRPV4	bronchial epithelial cells endothelium airway smooth muscle cells alveolar macrophages neutrophils myofibroblasts airway ganglia	inflammation, airway hyperreactivity and remodeling, bronchoconstriction, cough reflex	asthma, chronic obstructive pulmonary disease, pulmonary hypertension, lung fibrosis, lung edema, cystic fibrosis, cough, dry eyes	[16,56,68,69]
TRPC1	bronchial epithelial cells	airway remodeling	asthma	[18,51,70]
TRPС6	bronchial epithelial cells endothelium airway smooth muscle cells alveolar macrophages neutrophils fibroblasts myofibroblasts	inflammation, airway remodeling	cystic fibrosis, asthma	[51,71]
TRPM8	cold-sensitive afferents expressed in the upper and lower airways	inflammation, airway hyperreactivity and remodeling, cough reflex	asthma, thermosensation, pain sensation, lung cancers	[19,20,44,50,58,59,72,73,74,75,76,77]

**Table 4:** Predicted effects of missense variations of *TRPV1*, *TRPV4*, *TRPA1*, and *TRPM8* genes.

Gene	SNP	PROVEAN	SIFT	PolyPhen-2
*TRPV* *1*	rs222749 (p.Pro91Ser)	Neutral	Tolerated	Benign
	rs222747 (p.Met315Ile)	Neutral	Tolerated	Benign
	rs224534 (p.Thr469Ile)	Deleterious	Damaging	Benign
	rs17633288 (p.Thr505Ala)	Neutral	Tolerated	Benign
	rs8065080 (p.Ile585Val)	Neutral	Tolerated	Benign
*TRPV* *4*	rs3742030 (p.Pro19Ser)	Neutral	Tolerated	Benign
	rs531738577 (p.Tyr491Ser)	Deleterious	Damaging	Probably damaging
*TRPA1*	rs13268757 (p.Arg3Cys)	Neutral	Damaging	Benign
	rs16937976 (p.Arg58Thr)	Neutral	Tolerated	Benign
	rs920829 (p.Glu179Lys)	Deleterious	Tolerated	Probably damaging
	rs7819749 (p.Lys186Asn)	Neutral	Tolerated	Benign
	rs398123010 (p.Asn855Ser)	Neutral	Tolerated	Benign
	rs959976 (p.His1018Arg)	Neutral	Tolerated	Benign
*TRPM8*	rs13004520 (p.Arg247Thr)	Neutral	Damaging	Possibly damaging
	rs17868387 (p.Tyr251Cys)	Neutral	Tolerated	Probably damaging
	rs7593557 (p.Ser419Asn)	Neutral	Tolerated	Benign
	rs28902173 (p.Met462Thr)	Deleterious	Damaging	Probably damaging
	rs17862932 (p.Thr732Ile)	Deleterious	Damaging	Probably damaging
	rs28902201 (p.Asn821Ser)	Neutral	Tolerated	Benign

**Table 5:** Chemical ligands and physical stimuli.

TPR Channel	PDB Entry	Chemical Ligands and Physical Stimuli
TRPA1	6PQQ	[(2~{R})-1-[2-azanylethoxy(oxidanyl) phosphoryl]oxy-3-hexadecanoyloxy-propan-2-yl] (~{Z})-octadec-9-enoate
		1-palmitoyl-2-oleoyl-sn-glycero-3-phosphocholine
	6PQP	[(2~{R})-1-[2-azanylethoxy(oxidanyl) phosphoryl]oxy-3-hexadecanoyloxy-propan-2-yl] (~{Z})-octadec-9-enoate (BITS)
		N-benzylthioformamide
		1-palmitoyl-2-oleoyl-sn-glycero-3-phosphocholine
	6PQO	1-palmitoyl-2-oleoyl-sn-glycero-3-phosphocholine
		[(2~{R})-1-[2-azanylethoxy(oxidanyl) phosphoryl]oxy-3-hexadecanoyloxy-propan-2-yl] (~{Z})-octadec-9-enoate (JT010)
		2-chloro-N-[4-(4-methoxyphenyl)-1,3-thiazol-2-yl]-N-(3-methoxypropyl)acetamide
	3J9P	~{N}-[[2,2-bis(fluoranyl)-10,12-dimethyl-1,3-diaza-2l^{4}-boratricyclo[7.3.0.0^{3,7}]dodeca-4,6,9,11-tetraen-4-yl]methyl]ethanamide
	6V9V	Calcium ion Ca^2+^
	6V9X	-
	6V9W	Calcium ion Ca^2+^
	6V9Y	A 967079-
	6X2J	5-amino-1-[(4-bromo-2-fluorophenyl)methyl]-N-(2,5-dimethoxyphenyl)-1H-1,2,3-triazole-4-carboxamide (GNE551)
	N/A	Acrolein (in cigarette smoke), Acetaminophen, paracetamol, apomorphine, auranofin, oxaliplatin, etodolac, H2S, urea, chlorpromazine, desflurane, hypochlorite hydrogen peroxide H_2_O_2_, menthol, chloroquine, polycyclic aromatic hydrocarbons, сold temperatures <8 °C
TRPV1	3SUI	Sulfate ion Calcium ion Ca^2+^
	3J5R	(6E)-N-[(4-Hydroxy-3-methoxyphenyl)methyl]-8-methylnon-6-enamide or capsaicin
	3J5Q	Resiniferatoxin (RTX)
		Double knot toxin (DxTx)
	3J5P	-
	5IRX	Resiniferatoxin (RTX)
		Double knot toxin (DxTx)
		(4R,7S)-4-hydroxy-N,N,N-trimethyl-4,9-dioxo-7-[(pentanoyloxy)methyl]-3,5,8-trioxa-4lambda~5~-phosphatetradecan-1-aminium
		(2S)-2-(acetyloxy)-3-{[(R)-(2-aminoethoxy) (hydroxy) phosphoryl]oxy}propyl pentanoate
		(2S)-3-{[(S)-(2-aminoethoxy)(hydroxy) phosphoryl]oxy}-2-(hexanoyloxy)propyl hexanoate
	5IRZ	(2S)-1-{[(R)-hydroxy{[(1R,2R,3S,4S,5S,6S)-2,3,4,5,6-pentahydroxycyclohexyl] oxy}phosphoryl]oxy}-3-(pentanoyloxy)propan-2-yl decanoate
		(4R,7S)-4-hydroxy-N,N,N-trimethyl-4,9-dioxo-7-[(pentanoyloxy)methyl]-3,5,8-trioxa-4lambda~5~-phosphatetradecan-1-aminium
		(2S)-3-{[(S)-(2-aminoethoxy)(hydroxy) phosphoryl]oxy}-2-(hexanoyloxy)propyl hexanoate
	5IS0	Capsazepine
	3J9J	-
	N/A	Noxious heat >42 °C Pain
TRPV2	6OO3	Resiniferatoxin
	6OO7	Resiniferatoxin
	5AN8	-
	5HI9	-
	6BWJ	Resiniferatoxin
		Calcium ion Ca^2+^
	6BWM	Calcium ion Ca^2+^
	N/A	noxious heat >42 °C pain
TRPV3	LGP	(2S)-3-(hexadecanoyloxy)-2-[(9Z)-octadec-9-enoyloxy]propyl 2-(trimethylammonio)ethyl phosphate
		Diundecyl phosphatidyl choline
	6MHO	
	6MHS	
	6MHV	
	6MHX	2-Aminoethoxydiphenyl borate (2-APB)
	6MHW	
	6UW4	[(2~{R})-1-[2-azanylethoxy(oxidanyl) phosphoryl]oxy-3-hexadecanoyloxy-propan-2-yl] (~{Z})-octadec-9-enoate
		Sodium ion Na^+^
	6UW6	[(2~{R})-1-[2-azanylethoxy(oxidanyl) phosphoryl]oxy-3-hexadecanoyloxy-propan-2-yl] (~{Z})-octadec-9-enoate
	6UW9	
	6UW8	
	6DVY	2-aminoethyl diphenylborinate (2-APB)
	6DVZ	2-aminoethyl diphenylborinate (2-APB)
	6DVW	-
	6PVM	Sodium ion Na^+^
	6PVL	Sodium ion Na^+^
	6PVO	-
	6PVN	Sodium ion Na^+^
	6PVQ	-
	6PVP	Sodium ion Na^+^
	6OT5	2-aminoethyl diphenylborinate (2-APB)
	6OT2	-
TRPV4	6C8F	Cesium ion Cs+
	6C8H	Gadolinium atom Gd^+^
	6C8G	Barium ion Ba^2+^
	6BBJ	-
TRPC5	6YSN	7-[(4-chlorophenyl)methyl]-3-methyl-1-(3-oxidanylpropyl)-8-[3-(trifluoromethyloxy)phenoxy]purine-2,6-dione
TRPC6	6CV9	
	5YX9	
	6UZ8	2-[[(2~{S})-2-decanoyloxy-3-dodecanoyloxy-propoxy]-oxidanyl-phosphoryl]oxyethyl-trimethyl-azanium
		Agonist AM-0883
		Cholesterol hemisuccinate
		(5-chloro-1′H-spiro[indole-3,4′-piperidin]-1′-yl)[(2R)-2,3-dihydro-1,4-benzodioxin-2-yl]methanone
	6UZA	[(2~{S})-1-[2-azanylethoxy(oxidanyl) phosphoryl]oxy-3-octanoyloxy-propan-2-yl] octadecanoate
		Antagonist AM-1473
		Cholesterol hemisuccinate
		2-[[(2~{S})-2-decanoyloxypropoxy]-oxidanyl-phosphoryl]oxyethyl-trimethyl-azanium
		4-({(1R,2R)-2-[(3R)-3-aminopiperidin-1-yl]-2,3-dihydro-1H-inden-1-yl}oxy)benzonitrile
	7A6U	Zinc ion Zn^2+^
TRPM8	6NR4	Icilin, PI(4,5)P2 Calcium ion Ca^2+^
	6NR2	(2S)-1-{[(R)-hydroxy{[(1R,2R,3S,4R,5R,6S)-2,3,6-trihydroxy-4,5-bis(phosphonooxy) cyclohexyl]oxy}phosphoryl]oxy}-3-(octadecanoyloxy)propan-2-yl icosa-5,8,11,14-tetraenoate
		Menthol analog WS-12 and PI(4,5)P2)
		(1R,2S,5R)-N-(4-methoxyphenyl)-5-methyl-2-(propan-2-yl)cyclohexane-1-carboxamide
	6NR3	(2S)-1-{[(R)-hydroxy{[(1R,2R,3S,4R,5R,6S)-2,3,6-trihydroxy-4,5-bis(phosphonooxy)cyclohexyl]oxy}phosphoryl]oxy}-3-(octadecanoyloxy)propan-2-yl icosa-5,8,11,14-tetraenoate
		Icilin
		Calcium ion Ca^2+^
	6BPQ	-
	6O6A	Cholesterol hemisuccinate
		Sodium ion Na^+^
	6O77	Cholesterol hemisuccinate
		Calcium ion Ca^2+^
	6O6R	(1R)-2-{[(S)-(2-aminoethoxy)(hydroxy) phosphoryl]oxy}-1-[(heptanoyloxy)methyl]ethyl octadecanoate
		Cholesterol hemisuccinate
		N-(3-aminopropyl)-2-[(3-methylphenyl)methoxy]-N-[(thiophen-2-yl)methyl]benzamide
		Undecane
		Sodium ion Na^+^

**Table 6:** Pharmacological modulators for TRPA1, TRPV1, and TRPM8.

TRP Channels	Antagonists	References
TRPА1	GRC17536 HC-03003 CB189625 AP-18 Compound A HC030031	[79,107,108,109,110,111,112,113,114,115,116]
TRPV1	JNJ17203212 Capsazepine 5IS0 (Structure of TRPV1 in complex with capsazepine, determined in lipid nanodisc) JNJ39729209 XEN-DO501 SB705948 SB-705498 PF-04065463 SB-366791	[117,118,119,120,121,122,123]
TRPM8	AMTB N-arachidonyl dopamine Anandamide JNJ4187666 BCTC	[72,124,125,126,127,128]

## Data Availability

No datasets were generated during the study, statement is not necessary.
